# A Synthetic View on Haedoxans and Related Neolignans From *Phryma leptostachya*

**DOI:** 10.3389/fchem.2020.00460

**Published:** 2020-06-17

**Authors:** Yang Chen, Shu Xiao, Jian Huang, Wei Xue, Shuzhong He

**Affiliations:** ^1^Laboratory Breeding Base of Green Pesticide and Agricultural Bioengineering, Key Laboratory of Green Pesticide and Agricultural Bioengineering Ministry of Education, Guizhou University, Guiyang, China; ^2^Guizhou Engineering Laboratory for Synthetic Drugs, School of Pharmaceutical Sciences, Guizhou University, Guiyang, China

**Keywords:** haedoxans, neoligans, *Phryma leptostachya*, insecticidal activity, natural products

## Abstract

Haedoxans are a series of sesquilignan natural products isolated from the traditional insecticidal plant *Phryma leptostachya*. Given their significant insecticidal activity, haedoxans and related analogs have been considered as potential agents for plant defense. Moreover, these compounds also exhibit promising antifungal, antibacterial, and anticancer activities. The present paper is a review of the structure, biological activity, and chemical synthesis of naturally occurring haedoxan-like molecules.

## Introduction

*Phryma leptostachya* is a perennial herb that is widespread in nature (Lee et al., 2002; Park et al., 2005; Endo and Miyauchi, 2006; Li et al., 2019a,b; Xu et al., 2019). In Chinese culture, the plant has been used as a traditional Chinese medicine to treat inflammatory diseases, such as itching, allergic dermatitis, and gout (Jung et al., 2013). In East Asia, *P. leptostachya* has also been traditionally used as a natural insecticide (Taniguchi and Oshima, 1972a,b; Ishibashi and Taniguchi, 1998; Xiao et al., 2012a; Jung et al., 2013), for instance being used to repel mosquitos and flies in the southwest district of China (Chen et al., 2012). As a result, the secondary metabolites isolated from *P. leptostachya* have drawn much attention.

**Figure 1:**
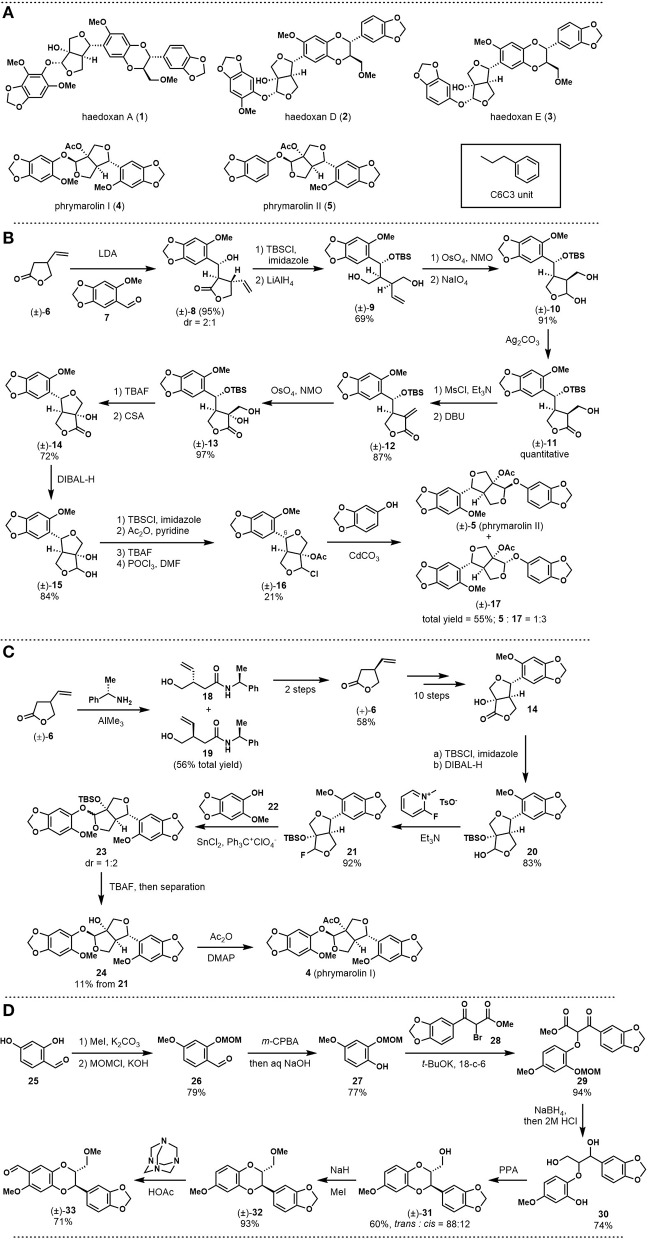
**(A)** Structures of haedoxans and phrymarolins; **(B)** Taniguchi's first total synthesis of (±)-phrymarolin II; **(C)** Taniguchi's total synthesis of (+)-phrymarolin I; **(D)** Taniguchi's synthesis toward (±)-haedoxan A, D, and E: fragment preparation.

Previous phytochemical investigations showed that this plant is rich in lignans. Among them, (+)-haedoxan A (**1**, see Figure 1A), isolated in 1989 by Taniguchi, represents the major insecticidal ingredient (Taniguchi et al., 1989; Yamaguchi and Taniguchi, 1992a; Seo and Park, 2012). Structurally, this natural product is a sesquilignan, that is, a trimer of C6C3 units (*n*-propyl benzene). The skeleton features a furofuran core and a dioxane core with six stereogenic centers. Haedoxan D (**2**) and E (**3**) are from the same natural product family, which structurally differs from haedoxan A (**1**) at one of the aromatic rings. (+)-Phrymarolin I (**4**) and II (**5**) are also important ingredients of *P. leptostachya* extracts. As neolignans, these two compounds are phenylpropianoid dimers that share the same furofuran core with haedoxans.

Haedoxans exhibit excellent insecticidal activity against several insects, such as *Musca domestica* [Culex pipiens pallens (Xiao et al., 2012b)] and *Mythimna separata* (Xiao et al., 2012a). It is noteworthy that the insecticidal activity of Haedoxan A (**1**) is comparable to that of the commercial synthetic pyrethroids (Taniguchi et al., 1989; Hu et al., 2016). (+)-Phrymarolins I (**4**) and II (**5**) also show considerable synergistic activities with pyrethrin and carbamate pesticides (Park et al., 2005). Accordingly, haedoxans and phrymarolins could be used as the main insecticidal ingredients in new botanical pesticides. In addition, the potential utilities of these natural products as lead compounds in pesticide discovery are also attractive.

To date, Haedoxan A (**1**) has only been found in the root of *P. leptostachya* at very low concentration (from 0.004 to 0.009%). Although two total syntheses of headoxan A (**1**) have been reported by Taniguchi and Ishibashi, over 20 synthetic steps are needed to achieve a natural product with moderate selectivities (Ishibashi and Taniguchi, 1989, 1998). As a result, the availability of haedoxan A (**1**) is the main obstacle in its commercialization process. To address this problem, new synthetic routes for headoxans with high efficiencies and stereoselectivities are needed.

## Synthetic Studies Toward Haedoxan-like Molecules

In this review, we focus on the chemical synthesis of haedoxans and some closely related natural products such as phrymarolin I (**4**) and II (**5**). While a number of synthetic studies on the lignan family have been reported, there have been limited reports on the synthesis of haedoxans. Since a phenylpropanoid trimer bears six stereogenic centers, haedoxans are the most structurally complex members in this family. It is noteworthy that besides the four contiguous stereocenters, the two chiral carbons that are remote from the furofuran core might also be a significant synthetic challenge due to stereocorrelation problems in the fragment coupling process. As a result, it would be extremely difficult to control steroselectivites in the total synthesis of haedoxans.

In 1986, Ishibashi and Taniguchi reported the synthesis of (±)-phrymarolin II (**5**), which represents a pioneering study on the chemical synthesis of haedoxan-like natural products (Ishibashi and Taniguchi, 1986). As shown in Figure 1B, the authors started their synthesis with an aldol reaction between lactone **6** and benzaldehyde **7** to build the left fragment of phrymarolin II (**5**). The adduct **8** was protected with a TBS group, and the lactone was then reduced with LiAlH_4_ to afford diol **9**. After Upjohn dihydroxylation and oxidative cleavage with NaIO_4_, diol **9** was converted to semiacetal **10**, which possesses one of the two tetrahydrofurans in the central fragment of the natural product. The semiacetal was then oxidized to corresponding lactone (**11**) with a quantitative yield. Once the lactone was established, a two-step reaction sequence was carried out to realize a β-elimination process. The resulting α, β-unsaturated lactone **12** was then oxidized with Upjohn dihydroxylation to give diol **13** at 97% yield. After deprotection of the TBS group with TBAF, the second tetrahydrofuran ring and the C6 stereocenter were established through an acid-promoted etherification reaction. The product **14** was treated with DIBAL-H to reduce the lactone moiety, and the newly formed diol was differentiated within four steps to afford chloride **16**. Finally, a CdCO_3_ catalyzed substitution successfully introduced the right fragment to give (±)-phrymarolin II (**5**) with its stereoisomer **17** in a ratio of 1:3 (55% total yield).

In 1988, Ishibashi and Taniguchi improved the previous synthetic route and reported the total synthesis of (+)-phrymarolin I [**4**, (Ishibashi and Taniguchi, 1988)]. This asymmetric synthesis commenced with the preparation of the optically pure (+)-**4**. As shown in Figure 1C, aminolysis of (±)-**6** with (*S*)-1-phenylethanamine gave two diastereoisomers that could be separated through chromatography. Then, hydrolysis of **18** followed by lactonization afforded (+)-**6** at 58% yield. This chiral starting material was subjected to the above synthetic route to give **14** in an asymmetric fashion. Different from the previous synthesis, **14** was first protected by the TBS group and then reduced to lactol **20**, which was then fluorinated to set the stage for the subsequent fragment coupling. In the next event, phenol **22** was introduced in the presence of SnCl_2_ and trityl perchlorate to provide the coupling product **23** with a diastereomeric ratio of 1:2, favoriting the undesired diastereomer. The mixed products were desilylated and separated by preparative TLC to afford **24** in 11% yield over two steps. Finally, acylation of **24** provided the desired natural product (+)-phrymarolin I (**4**).

One year later, Ishibashi and Taniguchi applied their developed synthetic route to the total synthesis of (±)-haedoxan A (**1**), D (**2**), and E [**3**, (Ishibashi and Taniguchi, 1989)]. As shown in Figure 1D, the preparation of the benzodioxane fragment **33** is not trivial. Their synthesis commenced with selective methylation and MOM protection of the benzaldehyde **25**. After Dakin oxidation and hydrolysis, the resultant phenol **27** was etherificated with bromide **28** to give **29** as the coupling product. Global reduction with NaBH_4_ was followed with acid promoted deprotection to generate **30** at 74% yield. A PPA-mediated cyclization was then introduced to build the dioxane ring. Finally, methylation and selective formylation provided the desired aromatic fragment (±)-**33**.

**Figure 2:**
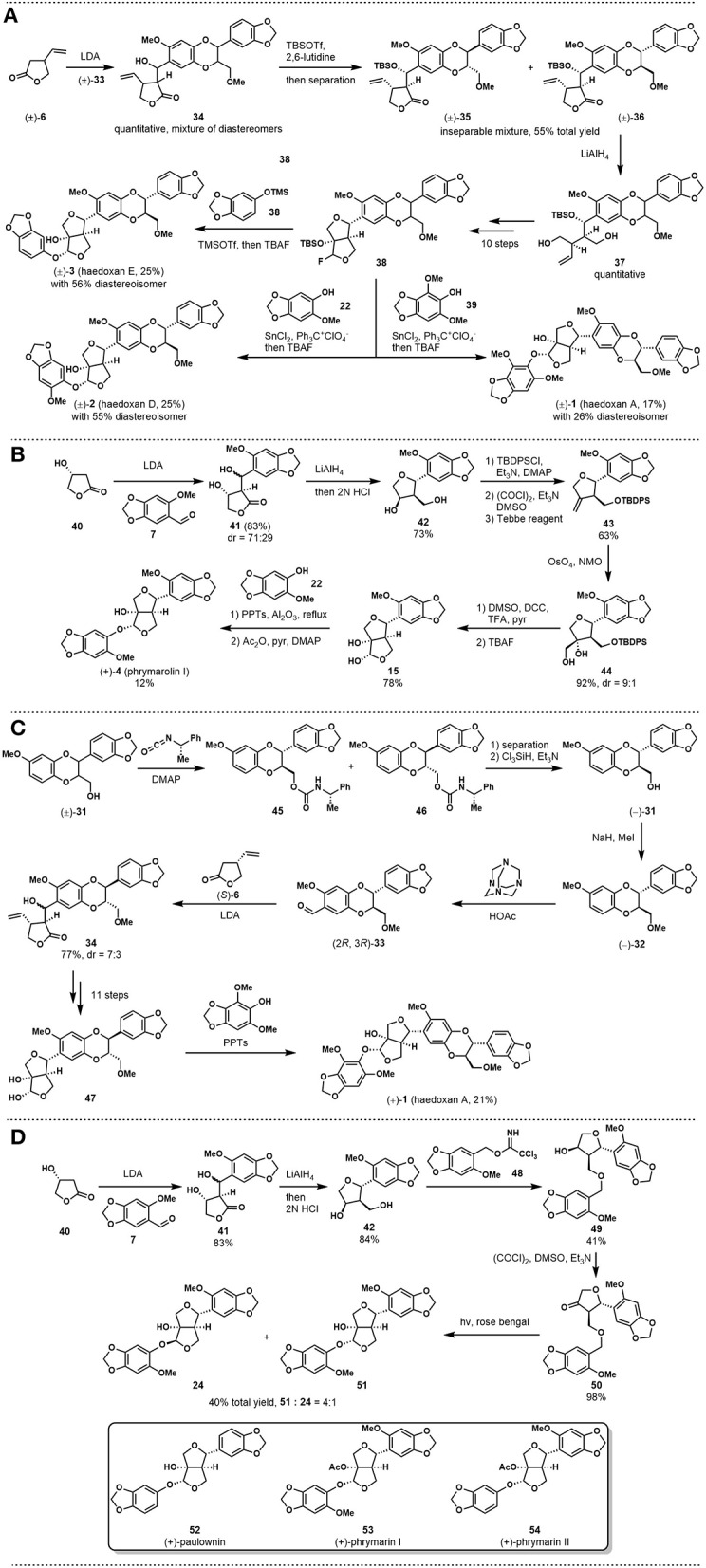
**(A)** Ishibashi and Taniguchi's total synthesis of (±)-haedoxan A, D, and E; **(B)** Taniguchi and Ishibashi's asymmetric synthesis of (+)-phrymarolin I; **(C)** Ishibashi and Taniguchi's asymmetric synthesis of (+)-haedoxan A; **(D)** Ishibashi's concise synthesis of (+)-paulownin, (+)-phrymarin I, and (+)-phrymarin II.

With the above fragment in hand, the authors followed their previous synthesis to carry out an aldol reaction between lactone **6** and aldehyde **33**, as shown in Figure 2A. However, although the reaction worked well, product **34** was obtained as a mixture of diastereomers due to stereochemical correlation issues. The adduct was protected with the TBS group and then purified by chromatography to afford an inseparable mixture of **35** and **36** at 55% total yield. The mixture was then submitted to the known synthetic route to give fluoride **38** within 11 steps. With this key intermediate, haedoxin A (**1**), D (**2**), E (**3**) were synthesized in diastereoselective fashion.

In the following decade, Taniguchi and coworkers applied their strategy to synthesizing a series of lignan analogs to explore potential insecticidal compounds (Yamaguchi and Taniguchi, 1991, 1992a,b,c; Yamaguchi et al., 1992a,b). A significant improvement of the synthetic strategy was published by Okazaki et al. (1997). In this report, the authors developed a concise synthetic route toward (+)-phrymarolin I (**4**). As shown in Figure 2B, the synthesis commenced with an aldol reaction using chiral lactone **40** as the nucleophile, which could be easily prepared from (*R*)-malate. After reductive opening of the lactone ring, the tetraol intermediate was treated with HCl solution to close the tetrahydrofuran ring and give **42**. Then, a three-step reaction sequence, including alcohol protection, Swern oxidation, and Tebbe olefination, was used to prepare alkene **43**. Diastereoselective dihydroxylatoin followed by Pfitzner-Moffatt oxidation and desilylation provided chiral lactol **15** at a good yield. The key intermediate that had been used in the authors' synthesis of phrymarolin II (**5**), **15**, was subjected directly to an acid-promoted replacement reaction with phenol **22** to afford the coupling product with desired stereochemistry at 15% yield. Then, a simple acylation reaction of the above product completed the asymmetric total synthesis of (+)-phrymarolin I (**4**).

In 1998, Ishibashi and Taniguchi reported their asymmetric synthesis of (+)-haedoxin A (**1**, Ishibashi and Taniguchi, 1998). While the core strategy followed the concept of Taniguchi's previous synthesis, this new synthesis featured the use of chiral synthons to avoid stereochemical correlation problems. As shown in Figure 2C, chiral compound **33** was first prepared *via* an optical resolution strategy from (±)-**31**. This synthon was coupled with another chiral building block (*S*)-**6** to give **34** as the adduct. Key intermediate **47** was then prepared through the known reaction sequence to set the stage for the last coupling. Instead of halogenation, the authors used the same key reaction in their synthesis of (+)-phrymarolin I (**4**) to install the phenol fragment directly on the lactol. This reaction provided the desired natural product, (+)-haedoxan A (**1**), at 21% yield.

In 2001, Ishibashi published a synthesis of (+)-paulownin, (+)-phrymarin I, and (+)-phrymarin II (Ishibashi et al., 2001). The report featured an elegant photochemical reaction that was developed by Kraus in 1990 (Kraus and Chen, 1990). As shown in Figure 2D, tetrahydrofuran intermediate **42** was synthesized through the procedures reported in Ishibashi's (+)-phrymarolin I (**4**) synthesis (Figure 2B). A coupling reaction between alcohol **42** and benzyl trichloroacetimidate **48**, followed by a Swern oxidation, provided the key intermediate **50** at 98% yield. Ketone **50** was then submitted to the photochemical condition developed by Kraus and Chen. In this event, a new C-C bond was formed between the irradiated benzylic position and the furan carbonyl in a diastereoselective manner to give furofuran **51** and **24** at 40% total yield. Finally, **51** was transformed into the desired natural product (+)-phrymarin I through a simple acylation reaction. With this concise synthetic route, the authors also completed the synthesis of (+)-paulownin and (+)-phrymarin II. It is noteworthy that compound **24** could serve as a key intermediate in the synthesis of (+)-phrymalorin I. However, the diastereoselectivity of the key reaction did not favor this intermediate.

## Summary and Further Prospects

Haedoxans and related neolignans are a family of natural insecticidal products with prominent potential applications. The main problem with the insecticide research process is the availability of sufficient samples. This review detailed the synthetic efforts toward haedoxans and phrymarolins in the past three decades. While these syntheses represent pioneering investigations on this topic, we are expecting new syntheses of haedoxans with higher efficiency, higher stereoselectivities, better step economy and redox economy, and more environmentally friendly procedures. This review may shed some light to guide future synthetic efforts on haedoxans.

## Author Contributions

YC collected and organized all articles in the literature regarding haedoxans and related neolignans from *Phryma Leptostachya*, reviewing the abstract, introduction, details of haedoxan syntheses, and summary and further prospects of each. SX helped check all of the figures and references. JH and WX reviewed all articles in the literature and participated in significant discussions. SH reviewed the synthetic efforts toward haedoxans and phrymarolins in the past three decades and summed them up in reaction figures.

## Conflict of Interest

The authors declare that the research was conducted in the absence of any commercial or financial relationships that could be construed as a potential conflict of interest.

## References

[B1] Chen C. Zhu H. Zhao D. Deng J. (2012). Lignans from *Phryma leptostachya* L. Helvetica Chimica Acta 95, 333–338. 10.1002/hlca.201100311

[B2] Endo Y. Miyauchi T. (2006). Thermonasty of young main stems of *Phryma leptostachya (Phrymaceae)*. J. Plant Res. 119, 449–457. 10.1007/s10265-006-0007-6 16896529

[B3] Hu Z. Du Y. Xiao X. Dong K. Wu W. (2016). Insight into the mode of action of Haedoxan A from *Phryma leptostachya*. Toxins 8, 53/51-53/12. 10.3390/toxins8020053 26907348 PMC4773806

[B4] Ishibashi F. Hayashita M. Okazaki M. Shuto Y. (2001). Improved procedure for the enantiometric synthesis of 1-Hydroxy/acetoxy-2,6-diaryl-3,7-dioxabicyclo[3.3.0]octane Lignans: Total Syntheses of (+)-Paulownin, (+)-Phrymarin I and (+)-Phrymarin II. Biosci. Biotech. Biochem. 65, 29–34. 10.1271/bbb.65.29 11272842

[B5] Ishibashi F. Taniguchi E. (1986). Synthesis of (±)-Phrymarolin II and Its Stereoisomers. Agric. Biol. Chem. 50, 3119–3125. 10.1080/00021369.1986.10867871

[B6] Ishibashi F. Taniguchi E. (1988). Synthesis and absolute configuration of the acetalic lignan (+)-Phrymarolin I. Bull. Chem. Soc. Jpn. 61, 4361–4366. 10.1246/bcsj.61.4361

[B7] Ishibashi F. Taniguchi E. (1989). Synthesis of (±)-Haedoxan A, D, E and their stereoisomers. Agric. Biol. Chem. 53, 1565–1573. 10.1271/bbb1961.53.1565

[B8] Ishibashi F. Taniguchi E. (1998). Synthesis and absolute configuration of the insecticidal sesquilignan (+)-Haedoxan A. Phytochemistry 49, 613–622. 10.1016/S0031-9422(98)00270-2

[B9] Jung H. Cho Y. Lim H. Choi H. Ji D. Lim C. (2013). Anti-inflammatory, antioxidant, anti-angiogenic and skin whitening activities of *Phryma leptostachya* var. asiatica Hara extract. Biomol. Ther. 21, 72–78. 10.4062/biomolther.2012.059 24009862 PMC3762305

[B10] Kraus G. A. Chen L. (1990). A total synthesis of racemic paulownin using a type II photocyclization reaction. J. Am. Chem. Soc. 112, 3464–3466. 10.1021/ja00165a033

[B11] Lee S. Min B. Kho Y. (2002). Brine shrimp lethality of the compounds from *Phryma leptostachya* L. Arch. Pharm. Res. 25, 652–654. 10.1007/BF02976939 12433200

[B12] Li Y. Wang S. Aioub A. A. A. Qie X. Wu W. Hu Z. (2019b). Identification and analysis of full-length transcripts involved in the biosynthesis of insecticidal lignan (+)-haedoxan A in *Phryma leptostachya*. Ind. Crops Prod. 142:111868 10.1016/j.indcrop.2019.111868

[B13] Li Y. Wei J. Fang J. Lv W. Ji Y. Aioub A. A. A. Zhang J. Hu Z. (2019a). Insecticidal activity of four lignans isolated from *Phryma leptostachya*. Molecules 24:1976. 10.3390/molecules24101976 31121976 PMC6572576

[B14] Okazaki M. Ishibashi F. Shuto Y. Taniguchi E. (1997). Total synthesis of (+)-Phrymarolin I from (+)-malic acid. Biosci. Biotech. Biochem. 61, 660–663. 10.1271/bbb.61.660

[B15] Park I. I. Shin S. Kim C. Lee H. Choi W. Ahn Y. (2005). Larvicidal activity of lignans identified in *Phryma leptostachya* Var. asiatica roots against three mosquito species. J. Agric. Food Chem. 53, 969–972. 10.1021/jf048208h 15713007

[B16] Seo S. Park I. I. (2012). Larvicidal activity of medicinal plant extracts and lignan identified in *Phryma leptostachya* var. asiatica roots against housefly *(Musca domestica L.)*. Parasitol. Res. 110, 1849–1853. 10.1007/s00436-011-2709-5 22065063

[B17] Taniguchi E. Imamura K. Ishibashi F. Matsui T. Nishio A. (1989). Structure of the novel insecticidal sesquilignan, haedoxan A. Agric. Biol. Chem. 53, 631–643. 10.1080/00021369.1989.10869338

[B18] Taniguchi E. Oshima Y. (1972a). Phrymarolin-I, a Novel Lignan from *Phryma leptostachya* L. Agric. Biol. Chem. 36, 1013–1025. 10.1271/bbb1961.36.1013

[B19] Taniguchi E. Oshima Y. (1972b). Structure of Phrymarolin-II. Agric. Biol. Chem. 36, 1489–1496. 10.1080/00021369.1972.10860431

[B20] Xiao X. Hu Z. Ji Z. Shi J. Zhang J. Wei S. (2012a). Isolation, structure identification and bioactivity of active ingredients from Phryma leptostachya. Chin. J. Pestic. Sci. 14, 583–586. 10.3969/j.issn.1008-7303.2012.05.19

[B21] Xiao X. Hu Z. Shi B. Wei S. Wu W. (2012b). Larvicidal activity of lignans from *Phryma leptostachya* L. against Culex pipiens pallens. Parasitol. Res. 110, 1079–1084. 10.1007/s00436-011-2591-1 21858479

[B22] Xu W. Zhao P. Wang M. Liang Q. (2019). Naturally occurring furofuran lignans: structural diversity and biological activities. Nat. Prod. Res. 33, 1357–1373. 10.1080/14786419.2018.1474467 29768037

[B23] Yamaguchi S. Ishibashi F. Taniguchi E. (1992b). Insecticidal Activity of Sesquilignans with a 3-Aryl-6-mehoxy-2-mehoxymethyl-1,4-benzodioxanyl Group. Biosci. Biotech. Biochem. 56, 1760–1768. 10.1271/bbb.56.1760

[B24] Yamaguchi S. Nagata S. Taniguchi E. (1992a). Effect on insecticidal activity of substituents at the 1,4-benzodioxanyl moiety of haedoxan. Biosci. Biotech. Biochem. 56, 1193–1197. 10.1271/bbb.56.1193

[B25] Yamaguchi S. Taniguchi E. (1991). Synthesis and insecticidal activity of lignan analogs (I). Agric. Biol. Chem. 55, 3075–3084. 10.1080/00021369.1991.10867924

[B26] Yamaguchi S. Taniguchi E. (1992a). Synthesis and insecticidal activity of lignan analogs (III). Biosci. Biotech. Biochem. 56, 418–422. 10.1271/bbb.56.418 27320991

[B27] Yamaguchi S. Taniguchi E. (1992b). Synthesis and insecticidal activity of lignan analogs (II). Biosci. Biotech. Biochem. 56, 412–417. 10.1271/bbb.56.412 27320990

[B28] Yamaguchi S. Taniguchi E. (1992c). Influence on insecticidal activity of the 3-(3,4-Methylenedioxyphenyl) group in the 1,4-Benzodioxanyl Moiety of Haedoxan. Biosci. Biotech. Biochem. 56, 1744–1750. 10.1271/bbb.56.1744

